# Epidemiology and Reporting Characteristics of Systematic Reviews in Orthopedic Journals: A Meta-Epidemiological Study

**DOI:** 10.3390/jcm12227031

**Published:** 2023-11-10

**Authors:** Norio Yamamoto, Shunsuke Taito, Takanori Miura, Takashi Ariie, Yosuke Tomita, Hirofumi Ogihara, Daijo Shiratsuchi, Takashi Yorifuji, Yasushi Tsujimoto

**Affiliations:** 1 Department of Orthopedic Surgery, Hashimoto Hospital, Mitoyo 768-0103, Japan; 2 Department of Epidemiology, Graduate School of Medicine, Dentistry and Pharmaceutical Sciences, Okayama University, Okayama 700-8558, Japan; yorichan@md.okayama-u.ac.jp; 3 Scientific Research WorkS Peer Support Group (SRWS-PSG), Osaka 541-0043, Japan; shutaitou@gmail.com (S.T.); tmlucky8@gmail.com (T.M.); tks.ar1212@gmail.com (T.A.); ogihara36@gmail.com (H.O.);; 4 Division of Rehabilitation, Department of Clinical Practice and Support, Hiroshima University Hospital, Hiroshima 734-8551, Japan; 5 Department of Orthopedic Surgery, Akita Rosai Hospital, Odate 018-5604, Japan; 6 Department of Physical Therapy, School of Health Sciences at Fukuoka, International University of Health and Welfare, Okawa 831-8501, Japan; 7 Department of Physical Therapy, Faculty of Health Care, Takasaki University of Health and Welfare, Takasaki 370-0033, Japan; tomita-y@takasaki-u.ac.jp; 8 Division of Physical Therapy, Department of Rehabilitation, Faculty of Health Sciences, Nagano University of Health and Medicine, Nagano City 381-2227, Japan; 9 Graduate School of Health Sciences, Kagoshima University, Kagoshima 890-8544, Japan; shiratsuchi@health.nop.kagoshima-u.ac.jp; 10 Department of Physical Therapy, School of Health Sciences, Faculty of Medicine, Kagoshima University, Kagoshima 890-8544, Japan; 11 Departments of Health Promotion and Human Behavior, Kyoto University Graduate School of Medicine/School of Public Health, Kyoto 606-8501, Japan; 12 Oku Medical Clinic, Osaka 573-0164, Japan

**Keywords:** meta-analysis, systemic reviews, reporting guidelines, PRISMA, full search strategy

## Abstract

Systematic reviews (SRs) with complete reporting or rigorous methods can lead to less biased recommendations and decisions. A comprehensive analysis of the epidemiological and reporting characteristics of SRs in orthopedics is lacking. We evaluated 360 SRs, including 165 and 195 published in orthopedic journals in 2012 and 2022. According to the established reporting guidelines, we examined these SRs for key epidemiological characteristics, including focus areas, type of meta-analysis (MA), and reporting characteristics. Most SRs (71%) were therapy-related, with a significant proportion originating from authors in the USA, UK, and China. Pairwise MA was performed on half of the SRs. The proportion of protocol registrations improved by 2022 but remained low (33%). Despite a formal declaration of adherence to the reporting guidelines (68%), they were often not used and reported enough. Only 10% of the studies used full search strategies, including trial registries. Publication bias assessments, subgroup analyses, and sensitivity analyses were not even planned. The risk of bias assessment improved in 2022; however, the certainty of the evidence remained largely unassessed (8%). The use and reporting of standard methods in orthopedic SRs have remained suboptimal. Thus, authors, peer reviewers, journal editors, and readers should criticize the results more.

## 1. Introduction

Systematic reviews (SRs) collate and summarize available evidence on specific research questions [1]. SRs can estimate the magnitude of benefits and harm, which can be reliable [2]. Moreover, SRs with complete reporting or rigorous implementation methods can lead to less biased recommendations and decisions [3,4].

SRs have increased over the last 10 years, raising research waste concerns [2,5]. Meta-epidemiological studies have highlighted the mass production of SRs [2,6]. The epidemiology and reporting characteristics of SRs were reported in biomedical journals in 2016 [7]. The review revealed that many SRs were poorly performed. Thus, it reported and called for strategies to help reduce this avoidable waste in research. Following this review, the characteristics of SRs in specific fields, such as imaging and nutrition, were also underreported [8,9].

However, the epidemiology and reporting characteristics of SRs in orthopedics have not been sufficiently investigated. Most studies have been limited to reviews assessed for reporting or methodological quality (Preferred Reporting Items for Systematic Reviews and Meta-Analysis (PRISMA) or Assessment of Multiple Systematic Review) [10,11]. Therefore, the epidemiology of SRs in orthopedic journals, such as the prevalence, types of reviews (therapy, epidemiology, diagnosis, or prognosis reviews), types of search databases, and characteristics of primary studies, remains unknown.

This study aimed to describe and compare the epidemiological and reporting characteristics of SRs published in orthopedic journals in 2012 and 2022. Through rigorous evaluation, we aimed to elucidate the current state of orthopedic SRs and identify areas of improvement.

## 2. Materials and Methods

### 2.1. Study Design and Protocol

This cross-sectional meta-epidemiological study was registered with osf.io (https://osf.io/z63ye (accessed on 8 October 2023)), and we followed the reporting guidelines [12]. Publicly available data were used. Ethical approval and patient consent were not required for this study.

### 2.2. Study Search and Selection

We selected SRs published in orthopedic journals in 2012 and 2022. We followed the definition of SR by Moher et al. and PRISMA protocols (PRISMA-P) [13,14]. We included SRs published in English, including those on therapy, epidemiology (prevalence, etiology), diagnosis, and prognosis [7]. We excluded overviews of SRs (umbrella reviews), scoping reviews, and meta-epidemiological studies that provide data for methodological analysis following the original or secondary studies [7].

We defined the orthopedic journals based on the 2022 Thomas Institute of Science Information list [15], including 125 journals (Supplementary Materials S1). We included orthopedic SRs based on the following definitions: orthopedics is a branch of medicine that focuses on the care of the musculoskeletal system, consisting of the muscles, bones, joints, ligaments, and tendons. We defined publication dates in 2012 or 2022 as electronic pre-publications or traditional journal publication dates.

We searched for SRs published in orthopedic journals in 2012 and 2022 using MEDLINE (PubMed) with search strategies (Supplementary Materials S2) [16].

### 2.3. Screening

First, we randomly selected 500 studies (250 each in 2012 and 2022) using a random number generator in Microsoft Excel. Second, two of the six reviewers (NY, TM, TA, YTo, HO, and DS) independently screened the titles and abstracts and assessed their eligibility based on their full texts. Disagreements between both reviewers were resolved through discussion. Otherwise, a third reviewer acted as an arbiter (ST or YTs).

### 2.4. Data Extraction

Two of the six reviewers independently extracted data from the included studies using a standardized data collection form. We collected the epidemiological and reporting characteristics based on previous studies on the epidemiology and reporting of SRs [7] and the PRISMA 2020 statement [17] (Supplementary Materials S3).

The epidemiological characteristics in the SRs were as follows: journal name, journal impact factor (JIF) (2021) [15], number of authors, country of corresponding author, focus of review (therapy (treatment, prevention), epidemiology (prevalence, etiology), diagnosis, prognosis) [7], SR category (completely new (mentioning that it is a completely new SR), update of prior SR, newer scope than prior SR (e.g., new patients, intervention or outcomes reviewed), higher quality than prior SR, limitations of primary studies only (no mention of prior SR, and only mention of limitations of primary studies), others) [18], anatomical location, common International Classification of Diseases 11th Revision (ICD-11) codes, number of included studies, number of included participants, economics assessment (i.e., costs) considered, meta-analysis (MA) performed, and number of studies included in the largest Mas.

The conducting and reporting characteristics in the SRs were as follows: SR protocol registration (e.g., PROSPERO) mentioned, reporting guideline (e.g., PRISMA) mentioned, Cochrane handbook used, inclusion/exclusion criteria reported, number of databases searched (without trial registry), trial registry (e.g., ClinicalTrials.gov) searched, all identified studies screened by at least two authors, all data extracted by at least two authors, unpublished data acquired from original authors, study risk of bias/quality assessment by at least two authors, study risk of bias (RoB)/quality assessment tool used (e.g., RoB2), number of outcomes stated in the method, primary outcome stated, statistical significance of effect estimate for primary outcome, magnitude of heterogeneity (I^2^) in the MAs for primary outcome, Grading of Recommendations, Assessment, Development, and Evaluations (GRADE) assessment reported in a summary of findings table or text, proportion of certainty of evidence through GRADE assessment, risk of publication bias assessed (or intent to assess), subgroup analysis, sensitivity analysis, presence of conflict of interest (COI), and presence of funding.

### 2.5. Statistical Analysis

We summarized the data as frequency (number, %) for categorical data or median and interquartile range (IQR) for continuous data. We compared the epidemiological and reporting characteristics of SRs published in 2012 and 2022. Regarding exploratory analyses, we calculated odds ratios (95% confidence intervals (CI)) for the epidemiological and reporting characteristics of SRs published in 2022 (SRs with versus without protocol registration and those with versus without self-reported use of the PRISMA statement in therapeutic SRs). When the epidemiological or reporting characteristics were continuous variables, they were converted into binary data using the median values as the cutoff. All analyses were performed using Stata/SE 17.0.

## 3. Results

**Figure 1:**
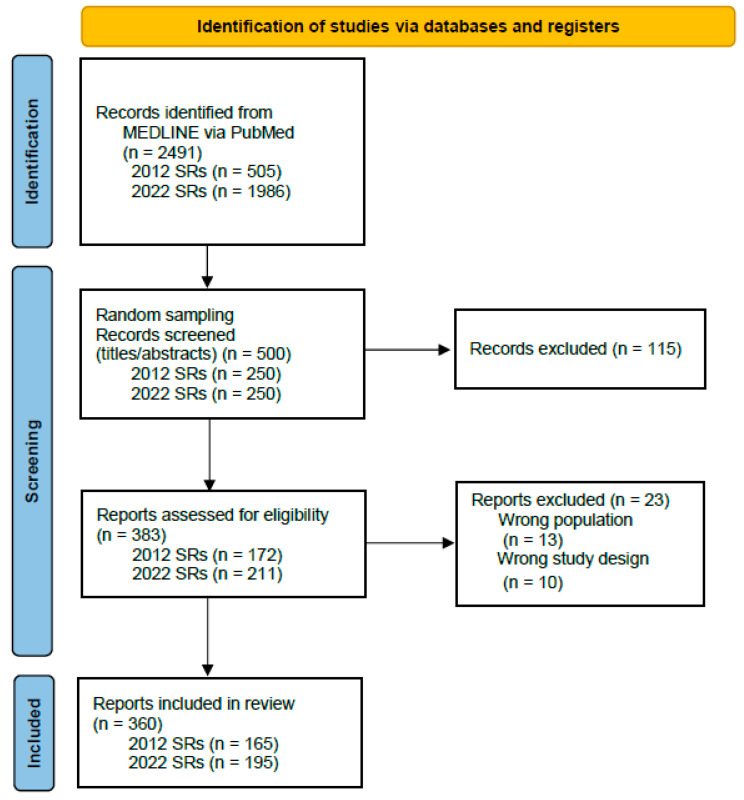
PRISMA 2020 flow diagram.

We identified 2491 SRs (Figure 1) and excluded 26 by screening for full texts (Supplementary Materials S4). Finally, we included 165 and 195 SRs published in 2012 and 2022, respectively, in the final analysis.

### 3.1. Epidemiological Characteristics

In total, 360 SRs were published in 80 journals, of which the highest number per journal was 15 (4%). SRs increased by 18% in 2022 compared to 2012. The JIF had a median of 3.2 (IQR: 2.6–4.4) in 2012, which declined to 2.8 (IQR: 2.5–3.5) in 2022 (Table 1 and Table 2). The corresponding authors were most frequently based in the USA, UK, or China and were responsible for 185/360 (51%) of the included SRs. Most SRs (256/360 (71%)) were classified as therapy and 39/360 (11%) as prognosis. In 2012, the SR category was dominated by the “completely new” type. However, the proportion decreased to about half by 2022. In all four types of SRs, “Completely new” increased, while “Limitations of primary studies only” decreased. SRs for infections and injuries increased in all four types of SRs in 2022, whereas diseases of the musculoskeletal system or connective tissue decreased by 2022. Few SRs (14/360 (4%)) considered these costs. No “empty reviews” (i.e., identified no eligible studies) were found. A meta-analysis was performed on 180/360 (50%) SRs, with a median of eight (IQR: 5–14) studies included in the largest meta-analysis in each SR. The pairwise MA was common both in 2012 and 2022. A network meta-analysis was not performed in 2012; however, it was 3% in 2022.

### 3.2. Conducting and Reporting Characteristics

We summarized the reporting characteristics of 165 and 189 SRs in 2012 and 2022, respectively, and those of the four types of SRs in Table 3 and Supplementary Materials S5. In 2022, six SRs were excluded from the reporting evaluation because they did not meet the inclusion criteria.

Protocol registration improved by 33% in 2022 compared to 2% in 2012, with the majority opting for registration with the International Prospective Register of Systematic Reviews (PROSPERO). Among the 2022 SRs, 7/56 (13%) were non-PROSPERO, of which two were International Platform of Registered Systematic Review and Meta-analysis Protocols, and one each was in the other registry. The authors used the reporting guidelines for 239/354 (68%) SRs. Among the SRs published in 2022 that used PRISMA-related reporting guidelines, PRISMA 2020 was used in only 23/153 (15%), whereas PRISMA 2009 was still used in 80/153 (52%). A few authors reported using Cochrane handbooks (56/354 (16%)). The number of databases was one for 27 SRs and two for 51 SRs in 2012 and 2022, respectively. A few authors searched the trial registry (26/354 (7%)). At least two authors screened 250/354 SRs (71%), whereas data extraction was performed by at least two authors in fewer SRs (40/354 (44%)). The percentage of unpublished data acquired from original authors in a few SRs (40/354 (11%)) was lower in 2022 than in 2012.

The risk of bias assessment by at least two authors improved slightly in 2022 compared to 2012. However, some authors of SRs published in 2022 did not report a risk of bias (32/189 (17%)). In SRs with MA, the risk of bias was assessed in 40/52 (77%) in 2012 and 74/76 (97%) in 2022. The primary outcome was more often specified in 2022 SRs 89/189 (47%). Among the SRs that reported an effect estimate for the primary outcome, the most common results were favorable and significant (56/128 (44%)). The SRs for therapy were more often significant (17% in 2012 and 22% in 2022). Most authors did not assess the magnitude of heterogeneity in MAs for the primary outcome (217/354 (61%)). A few authors reported the GRADE assessment (29/354 (8%)), and no improvement was observed in 2022. The certainty of evidence in MAs was very low (1–2%). Notably, most authors (approximately 80%) did not outline plans for assessing publication bias, subgroup analyses, or sensitivity analyses.

Protocol registration was significantly associated with the reporting characteristics: reporting guideline mentioned (odds ratio (OR): 11.4, 95% CI 4.5–2.92), trial registry searched (4.0, 1.8–9.1), screening by at least two authors (2.3, 1.2–4.6), risk of bias assessment tool (11.4, 4.5–29.2), and primary outcome stated (1.9, 1.1–3.2) (Supplementary Materials S6). Self-reported PRISMA use was significantly associated with reporting the risk of bias assessment tool used (308.4, 16.1–5904.5) (Supplementary Materials S7).

## 4. Discussion

We evaluated the characteristics of 165 and 195 SRs published in 2012 and 2022 in orthopedic journals. Most SRs focused on therapy and were conducted by authors from the USA, UK, and China. The SR category was mostly “Completely new” in 2012; however, the proportion decreased to about half by 2022. MA was performed in half of the SRs using pairwise MAs. The proportion of protocol registrations improved in 2022, but remained low. Only 10% of the studies used full search strategies, including trial registries. However, the heterogeneity in MAs has not yet been reported. Particularly, the assessment of publication bias, subgroup analysis, and sensitivity analysis was unplanned. The risk of bias assessment improved in 2022; nonetheless, the GRADE remained largely unassessed.

The epidemiological characteristics of SRs have varied over time. This tendency is consistent with the results of biomedical and imaging journals [7,8]. This can be attributed to the changing clinical practice and research trends, evolving technologies, surgical techniques, and research methods. Particularly in therapy, network MA will increase in addition to the traditional pairwise MA as more RCTs on new interventions are conducted in the future. In addition, the individual participant data MA is expected to increase as the number of studies that share data increases. Once the treatment effectiveness is clarified, the next step should be further research on cost-effectiveness as a health-economic decision.

Orthopedic SRs remained suboptimal in using and reporting the standard method. Our findings align with earlier results of poor reporting in SRs of non-orthopedic fields [8,9], suggesting that these issues are not unique to orthopedics and warrant broader attention. Important items underreported in previous studies, such as protocol registration, PRISMA guidelines, and GRADE, were also poorly described in orthopedic SRs. Only 33% of orthopedic SRs in 2022 were protocol-registered, lower than the proportion in medical journals generally (55.8%) [19]. In 2022, only 12% of the SRs used PRISMA 2020, indicating that the authors could not comply with the updated reporting guidelines. Only approximately 10% of SRs consider the certainty of evidence, similar to other fields [20,21]. Interpreting the results based on the certainty of evidence rather than focusing only on the statistical significance of the effect estimates is essential.

The exploratory analysis revealed that the self-reported use of PRISMA was unassociated with sufficient reporting. Therefore, the formal description of the guidelines in the manuscript was not reflected in the use and description [22]. Unfortunately, many journals that recommend reporting guidelines have incomplete reports [23].

We suggest some actions for improving the reporting of SRs. First, applying an electronic system powered by artificial intelligence during submission is an option to automatically check the minimum required reporting accuracy [24]. Second, authors should design a method to avoid underreporting by using a format at the time of the protocol [25]. Third, the journal should list the specific items that need to be described and the reporting guideline to be followed (e.g., PRISMA2020) in the submission guidelines. In addition, authors must submit the reporting guideline checklist. Finally, peer reviewers and editors should review and evaluate the SR according to the reporting checklist.

This review had several strengths. First, this was the first study to investigate the epidemiological and reporting characteristics of SRs in an orthopedic journal. The results are presented separately for each of the four SR focuses, owing to their diverse characteristics and analytical methods. Our results expand on existing knowledge by highlighting the specific shortcomings of orthopedic SRs and reaffirming the call for strategies to bolster the quality of orthopedic SRs. Second, two authors independently selected studies and extracted data to minimize the risk of measurement errors. Third, both groups of proportions were compared, ensuring a minimum of 100 SRs in each group, making 360 SRs sufficient for analysis.

This study had some limitations. First, there was a selection bias in the year of publication, studies published in the English language, and the single database search. Therefore, we may be unable to generalize our findings to SRs published in other years, languages, or databases. Second, these SRs (epidemiology, diagnosis, and prognosis) were based on a small sample size. Therefore, these proportions should be interpreted with caution.

In conclusion, the use and reporting of standard methods in orthopedic SRs have not improved significantly and remain suboptimal. Therefore, authors, peer reviewers, journal editors, and readers should be more critical of the results. Finally, strategies are needed to improve this modifiable reporting quality.

## Figures and Tables

**Table 1:** Epidemiological characteristics of systematic reviews in 2012 and 2022.

Characteristic	Category	Year	
		2012 (*n* = 165)	2022 (*n* = 195)
Journal impact factor (2021)	0.0–2.0	25 (15%)	22 (11%)
	2.1–5.0	101 (61%)	137 (70%)
	>5.0	27 (16%)	14 (7%)
	No impact factor	12 (7%)	22 (11%)
Number of authors		4 (3–6)	6 (4–7)
Country of corresponding author	USA	47 (28%)	42 (22%)
	UK	28 (17%)	20 (10%)
	China	20 (12%)	28 (14%)
Focus of review	Therapy	113 (68%)	143 (73%)
	Epidemiology (prevalence)	12 (7%)	19 (10%)
	Diagnosis	21 (13%)	7 (4%)
	Prognosis	19 (12%)	20 (10%)
SR category	Completely new	59 (36%)	31 (16%)
	Update of prior SR	14 (8%)	16 (8%)
	Newer scope than prior SR	19 (12%)	24 (12%)
	Higher quality than prior SR	21 (13%)	8 (4%)
	Limitations of primary studies only	52 (32%)	116 (59%)
Anatomical location	Upper limbs	24 (15%)	30 (15%)
	Lower limbs	77 (47%)	103 (53%)
	Spine	33 (20%)	43 (22%)
	Pelvis	5 (3%)	3 (2%)
Common ICD-11 codes	Certain infections and parasitic diseases	0 (0%)	11 (6%)
	Neoplasms	3 (2%)	2 (1%)
	Diseases of the nervous system	2 (1%)	2 (1%)
	Diseases of the circulatory system	2 (1%)	1 (1%)
	Diseases of the musculoskeletal system or connective tissue	139 (84%)	108 (55%)
	Injury, poisoning or certain other consequences of external causes	10 (6%)	67 (34%)
Number of included studies		12 (7–24)	14 (9–26)
Number of included participants		1040 (412–2414)	842 (394–1924)
Economics assessment (i.e., costs) considered		10 (6%)	4 (2%)
Meta-analysis performed	Single-arm MA	17 (10%)	23 (12%)
	Pairwise MA	56 (34%)	77 (39%)
	Network MA	0 (0%)	4 (2%)
	Individual participant data MA	1 (1%)	2 (1%)
	Not reported	91 (55%)	89 (46%)
Number of studies included in the largest meta-analysis		10 (7–14)	6 (4–11)

SR, systematic review; MA, meta-analysis; ICD, International Classification of Diseases. Data are presented as number (percent) or median (IQR).

**Table 2:** Comparison of the epidemiological characteristics of systematic reviews in orthopedic journals published in 2012 and 2022, stratified by focus of systematic reviews.

Characteristic	Category	Therapy			Epidemiology (Prevalence)			Diagnosis			Prognosis		
		2012 (*n* = 113)		2022 (*n* = 143)	2012 (*n* = 12)		2022 (*n* = 19)	2012 (*n* = 21)		2022 (*n* = 7)	2012 (*n* = 19)		2022 (*n* = 20)
Number of included SRs		113	↑	143	12	↑	19	21	↓	7	19	↑	20
Journal impact factor (2021)		2.9 (2.4–4.1)	↓	2.8 (2.4–3.5)	2.9 (2.4–4.4)	=	2.9 (2.7–3.4)	4.1 (2.7–6.6)	↓	2.7 (2.2–2.7)	3.2 (2.7–4.4)	↓	2.9 (2.6–4.1)
Number of authors		4 (3–6)	↑	6 (4–7)	3.5 (3–5)	↑	5 (5–6)	6 (4–7)	↓	5 (4–9)	5 (3–6)	=	5 (4–6.5)
Country of corresponding author	USA	32%	↓	19%	25%	↓	42%	14%	=	14%	26%	↓	25%
	UK	16%	↓	8%	25%	↓	11%	19%	↓	0%	16%	↑	20%
	China	16%	↑	17%	8%	↓	0%	5%	↑	29%	0%	↑	5%
SR category	Completely new	33%	↓	17%	50%	↓	11%	52%	↓	29%	26%	↓	15%
	Update of prior SR	9%	=	9%	8%	↓	0%	10%	↓	0%	5%	↑	15%
	Newer scope than prior SR	14%	↑	15%	0%	=	0%	0%	↑	14%	16%	↓	10%
	Higher quality than prior SR	14%	↓	5%	0%	=	0%	19%	↓	0%	5%	=	5%
	Limitations of primary studies only	30%	↑	55%	42%	↑	89%	19%	↑	57%	47%	↑	55%
Anatomical location	Upper limbs	15%	↑	17%	25%	↓	11%	14%	↓	0%	5%	↑	10%
	Lower limbs	48%	↑	55%	33%	↑	42%	43%	↓	29%	53%	↑	65%
	Spine	23%	↓	20%	25%	↑	32%	10%	↑	43%	11%	↑	15%
	Pelvis	2%	↓	1%	8%	↓	5%	0%	=	0%	11%	↓	0%
Common ICD-11 codes	Certain infections and parasitic diseases or certain other consequences of external causes	0%	↑	4%	0%	↑	5%	0%	↑	43%	0%	↑	5%
	Neoplasms	2%	↓	0%	0%	↑	5%	5%	↑	14%	0%	=	0%
	Diseases of the nervous system	2%	↓	1%	0%	=	0%	0%	=	0%	0%	=	0%
	Diseases of the circulatory system	1%	=	1%	0%	=	0%	5%	↓	0%	0%	=	0%
	Diseases of the musculoskeletal system or connective tissue	84%	↓	59%	83%	↓	47%	81%	↓	14%	89%	↓	45%
	Injury, poisoning or certain other consequences of external causes	6%	↑	34%	0%	↑	37%	5%	↑	29%	11%	↑	45%
Number of included studies		11 (7–18)	↑	13 (9–25)	14 (8–89)	↑	20 (9–42)	17 (9–31)	↓	11 (8–23)	15 (9–29)	↑	17 (13–28)
Number of included participants		768 (309–1707)	↑	780 (385–1764)	598 (365–2072)	↑	1244 (250–3197)	1374 (604–2281)	↓	1183.5 (525–1467)	23,030.5 (1839–37,635)	↓	4349 (1574–67,818)
Economics assessment (i.e., costs) considered		8%	↓	1%	NA		NA	NA		NA	NA		NA
Meta-analysis performed	Single-arm MA	4%	↑	9%	17%	↓	16%	33%	↑	43%	16%	↓	15%
	Pairwise MA	41%	↑	45%	8%	↑	16%	10%	↓	0%	37%	↑	50%
	Network MA	0%	↑	3%	NA		NA	NA		NA	NA		NA
	Individual participant data MA	1%	=	1%	0%	=	0%	0%	↑	57%	0%	=	0%
	Not reported	54%	↓	42%	75%	↓	68%	57%	↓	0%	47%	↓	35%
Number of studies included in the largest meta-analysis		8 (5–14)	=	8 (6–14)	12 (5–89)	↓	8.5 (6–12)	7 (7–9)	↑	13 (7–14)	7 (5–13)	↑	10 (6–12)

SR, systematic review; MA, meta-analysis; ICD, International Classification of Diseases; NA, not applicable. Data are presented as number (percent) or median (IQR). Direction of change in 2012 versus 2022 means as follows; ↑, increase; ↓, decrease; =, no change.

**Table 3:** Comparison of the conducting and reporting characteristics of systematic reviews in orthopedic journals published in 2012 and 2022.

Characteristic	Category	All (*n* = 354)	2012 (*n* = 165)		2022 (*n* = 189)
SR protocol registration (e.g., PROSPERO) mentioned	Not reported	288 (81%)	162 (98%)	↓	126 (67%)
	PROSPERO	57 (16%)	1 (1%)	↑	56 (30%)
Reporting guideline (e.g., PRISMA) mentioned	Not reported	139 (39%)	115 (70%)	↓	24 (13%)
	PRISMA 2009	108 (31%)	28 (17%)	↑	80 (42%)
	PRISMA 2020	23 (6%)	0 (0%)	↑	23 (12%)
	PRISMA extension and the other PRISMA-related	65 (18%)	15 (9%)	↑	50 (26%)
Cochrane handbook used		56 (16%)	29 (18%)	↓	27 (14%)
Inclusion/exclusion criteria reported		312 (88%)	133 (81%)	↑	179 (95%)
Number of databases searched (without trial registry)		3 (3–5)	3 (2–5)	=	3 (3–4)
Trial registry (e.g., ClinicalTrials.gov) searched		26 (7%)	9 (5%)	↑	17 (9%)
All identified studies screened by at least two authors		250 (71%)	93 (56%)	↑	157 (83%)
All data extracted by at least two authors		155 (44%)	57 (35%)	↑	98 (52%)
Unpublished data acquired from original authors		40 (11%)	30 (18%)	↓	10 (5%)
Study risk of bias/quality assessment by at least two authors		160 (45%)	70 (42%)	↑	90 (48%)
Study risk of bias/quality assessment tool used	Not reported	95 (27%)	63 (38%)	↓	32 (17%)
	Cochrane risk of bias tool	37 (10%)	20 (12%)	↓	17 (9%)
	Cochrane risk of bias tool 2.0	12 (3%)	0 (0%)	↑	12 (6%)
	MINORS	31 (9%)	1 (1%)	↑	30 (16%)
	Newcastle–Ottawa Scale	18 (5%)	7 (4%)	↑	11 (6%)
Number of outcomes stated in the method		3 (1–5)	3 (1–5)	=	3 (2–5)
Primary outcome stated		123 (35%)	34 (21%)	↑	89 (47%)
Statistical significance of effect estimate for primary outcome	Not reported	220 (62%)	110 (67%)	↓	110 (58%)
	Favorable and statistically significant	56 (16%)	21 (13%)	↑	35 (19%)
	Favorable and statistically nonsignificant	39 (11%)	17 (10%)	↑	22 (12%)
	Unfavorable and statistically significant	18 (5%)	9 (5%)	=	9 (5%)
	Unfavorable and statistically nonsignificant	15 (4%)	5 (3%)	↑	10 (5%)
	Direction of effect unclear	0 (0%)	0 (0%)	=	0 (0%)
Magnitude of heterogeneity (I^2^) in the MAs for primary outcome	Not reported	217 (61%)	113 (68%)	↓	104 (55%)
	<25%	49 (14%)	20 (12%)	↑	29 (15%)
	25 to <50%	23 (6%)	12 (7%)	↓	11 (6%)
	50 to <75%	24 (7%)	11 (7%)	↓	13 (7%)
	75% to 100%	41 (12%)	9 (5%)	↑	32 (17%)
GRADE assessment reported in a summary of findings table or text		29 (8%)	12 (7%)	↑	17 (9%)
Proportion of certainty of evidence via GRADE assessment	High certainty of evidence	4 (1%)	1 (1%)	↑	3 (2%)
	Moderate certainty of evidence	12 (3%)	5 (3%)	↑	7 (4%)
	Low certainty of evidence	15 (4%)	7 (4%)	=	8 (4%)
	Very low certainty of evidence	12 (3%)	7 (4%)	↓	5 (3%)
Risk of publication bias assessed (or intent to assess)	Not planned	288 (81%)	137 (83%)	↓	151 (80%)
	Formally assessed	62 (18%)	26 (16%)	↑	36 (19%)
	Not assessed but authors planned	4 (1%)	2 (1%)	=	2 (1%)
Subgroup analysis	Not planned	287 (81%)	134 (81%)	=	153 (81%)
	Formally assessed	60 (17%)	27 (16%)	↑	33 (17%)
	Not assessed but authors planned	5 (1%)	3 (2%)	↓	2 (1%)
Sensitivity analysis	Not planned	314 (89%)	144 (87%)	↑	170 (90%)
	Formally assessed	37 (10%)	18 (11%)	↓	19 (10%)
	Not assessed but authors planned	3 (1%)	3 (2%)	↓	0 (0%)
Presence of COIs	No	252 (71%)	101 (61%)	↑	151 (80%)
	Yes	71 (20%)	37 (22%)	↓	34 (18%)
	Not reported	31 (9%)	27 (16%)	↓	4 (2%)
Presence of funding	No	167 (47%)	59 (36%)	↑	108 (57%)
	Yes	97 (27%)	41 (25%)	↑	56 (30%)
	Not reported	90 (25%)	65 (39%)	↓	25 (13%)

SR, systematic review; MA, meta-analysis; GRADE, Grading of Recommendations, Assessment, Development, and Evaluations; COI, conflicts of interest; MINORS, methodological index for non-randomized studies; PRISMA; Preferred Reporting Items for Systematic Reviews and Meta-Analysis; NA, not applicable. Data are presented as number (percent) or median (IQR). Direction of change in 2012 versus 2022 means as follows; ↑, increase; ↓, decrease; =, no change.

## Supplementary Materials

The following supporting information can be downloaded at: https://www.mdpi.com/article/10.3390/jcm12227031/s1, Supplementary Materials S1: Included orthopedic journal lists. Supplementary Materials S2: MEDLINE (PubMed) search strategies. Supplementary Materials S3: Epidemiological and reporting characteristics. Supplementary Materials S4: List of studies excluded from this review and reasons for exclusion. Supplementary Materials S5: Comparison of the reporting characteristics of systematic reviews in orthopedic journals published in 2012 and 2022, stratified by focus of systematic reviews. Supplementary Materials S6: Odds ratio association reporting characteristics and protocol registration. Supplementary Materials S7: Odds ratio association reporting characteristics and self-reported use of PRISMA.
