# Supplementary material for: Epidemiology and Reporting Characteristics of Systematic Reviews in Orthopedic Journals: A Meta-Epidemiological Study
Source: J Clin Med. 2023 Nov 10;12(22):7031. doi: 10.3390/jcm12227031 (PMC10672058; doi:10.3390/jcm12227031)
Supplement: Supplementary file 1 [file jcm-12-07031-s001.zip › jcm-2674164-supplementary.pdf]

Supplementary Materials S1. Included orthopedic journal lists

Supplementary Materials S2. MEDLINE (PubMed) Search strategies

Supplementary Materials S3. Epidemiological and reporting characteristics

Supplementary Materials S4. List of studies excluded from this review and reasons for exclusion

Supplementary Materials S5. Comparison of the reporting characteristics of systematic reviews in orthopedic journals published in 2012 and 2022, stratified by focus of systematic reviews

Supplementary Materials S6. Odds ratio association reporting characteristics and protocol registration

Supplementary Materials S7. Odds ratio association reporting characteristics and self-reported use of PRISMA

Supplementary Materials S1. Included orthopedic journal lists

Journal	Search term in MEDLINE (PubMed) search strategy
ACTA CHIRURGIAE ORTHOPAEDICAE ET TRAUMATOLOGIAE CECHOSLOVACA	"Acta Chir Orthop Traumatol Cech"[jour]
ACTA ORTHOPAEDICA BELGICA	"Acta Orthop Belg"[jour]
ACTA ORTHOPAEDICA ET TRAUMATOLOGICA TURCICA	"Acta Orthop Traumatol Turc"[jour]
ACTA ORTHOPAEDICA	"Acta Orthop"[jour]
ACTA ORTOPEDICA BRASILEIRA	"Acta Ortop Bras"[jour]
ADVANCES IN EXPERIMENTAL MEDICINE AND BIOLOGY	"Adv Exp Med Biol"[jour]
ADVANCES IN ORTHOPEDICS	"Adv Orthop"[jour]
AMERICAN JOURNAL OF SPORTS MEDICINE	"Am J Sports Med"[jour]
ANNALS OF JOINT	"Ann Jt"[jour]
ARCHIVES OF BONE AND JOINT SURGERY	"Arch Bone Jt Surg"[jour]
ARCHIVES OF ORTHOPAEDIC AND TRAUMA SURGERY	"Arch Orthop Trauma Surg"[jour]

ARCHIVES OF OSTEOPOROSIS	"Arch Osteoporos"[jour]
ARCHIVES OF TRAUMA RESEARCH	"Arch Trauma Res"[jour]
ARTHROPLASTY	"Arthroplasty”[jour]
ARTHROSCOPY TECHNIQUES	"Arthrosc Tech"[jour]
ASIA PACIFIC JOURNAL OF SPORT MEDICINE ARTHROSCOPY REHABILITATION AND TECHNOLOGY	"Asia Pac J Sports Med Arthrosc Rehabil Technol"[jour]
ASIAN SPINE JOURNAL	"Asian Spine J"[jour]
BMC MUSCULOSKELETAL DISORDERS	"BMC Musculoskelet Disord"[jour]
BONE JOINT JOURNAL	"Bone Joint J"[jour]
BONE JOINT RESEARCH	"Bone Joint Res"[jour]
BONE JOINT OPEN	"Bone Jt Open"[jour]
BONE MARROW RESEARCH	"Bone Marrow Res"[jour]
BONEKEY REPORTS	"Bonekey Rep"[jour]
BRAZILIAN JOURNAL OF PHYSICAL THERAPY	"Braz J Phys Ther"[jour]
BULLETIN OF THE HOSPITAL FOR JOINT DISEASES	"Bull Hosp Jt Dis (2013)"[jour]
CARTILAGE	"Cartilage"[jour]
CHINESE JOURNAL OF TRAUMATOLOGY	"Chin J Traumatol"[jour]
CHIRURGIE DE LA MAIN	"Chir Main"[jour]
CLINICAL BIOMECHANICS	"Clin Biomech (Bristol, Avon)"[jour]
CLINICAL JOURNAL OF SPORT MEDICINE	"Clin J Sport Med"[jour]
CLINICAL MEDICINE INSIGHTS ARTHRITIS AND MUSCULOSKELETAL DISORDERS	"Clin Med Insights Arthritis Musculoskelet Disord"[jour]
CLINICAL ORTHOPAEDICS AND RELATED RESEARCH	"Clin Orthop Relat Res"[jour]
CLINICS IN ORTHOPEDIC SURGERY	"Clin Orthop Surg"[jour]
CLINICS IN PODIATRIC MEDICINE AND SURGERY	"Clin Podiatr Med Surg"[jour]
CLINICAL SPINE SURGERY	"Clin Spine Surg"[jour]
CONNECTIVE TISSUE RESEARCH	"Connect Tissue Res"[jour]

CURRENT ORTHOPAEDIC PRACTICE	"Curr Orthop Pract"[jour]
CURRENT REVIEWS IN MUSCULOSKELETAL MEDICINE	"Curr Rev Musculoskelet Med"[jour]
EFORT OPEN REVIEWS	"EFORT OPEN REVIEWS"[jour]
EUROPEAN CELLS MATERIALS	"Eur Cell Mater"[jour]
EUROPEAN JOURNAL OF ORTHOPAEDIC SURGERY AND TRAUMATOLOGY	"Eur J Orthop Surg Traumatol"[jour]
EUROPEAN SPINE JOURNAL	"Eur Spine J"[jour]
FOOT AND ANKLE CLINICS	"Foot Ankle Clin"[jour]
FOOT ANKLE INTERNATIONAL	"Foot Ankle Int"[jour]
FOOT ANKLE SPECIALIST	"Foot Ankle Spec"[jour]
FOOT AND ANKLE SURGERY	"Foot Ankle Surg"[jour]
GAIT POSTURE	"Gait Posture"[jour]
GERIATRIC ORTHOPAEDIC SURGERY REHABILITATION	"Geriatr Orthop Surg Rehabil"[jour]
GLOBAL SPINE JOURNAL	"Global Spine J"[jour]
HAND CLINICS	"Hand Clin"[jour]
HAND SURGERY REHABILITATION	"Hand Surg Rehabil"[jour]
HIP INTERNATIONAL	"Hip Int"[jour]
HSS JOURNAL	"HSS J"[jour]
INDIAN JOURNAL OF ORTHOPAEDICS	"Indian J Orthop"[jour]
INJURY INTERNATIONAL JOURNAL OF THE CARE OF THE INJURED	"Injury"[jour]
INTERNATIONAL JOURNAL OF SHOULDER SURGERY	"Int J Shoulder Surg"[jour]
INTERNATIONAL JOURNAL OF SURGERY OPEN	"Int J Surg Open"[jour]
INTERNATIONAL ORTHOPAEDICS	"Int Orthop"[jour]
ISOKINETICS AND EXERCISE SCIENCE	"Isokinet Exerc Sci"[jour]
JOURNAL OF THE AMERICAN ACADEMY OF ORTHOPAEDIC SURGEONS GLOBAL RESEARCH AND REVIEWS	"J Am Acad Orthop Surg Glob Res Rev"[jour]
JOURNAL OF THE AMERICAN ACADEMY OF ORTHOPAEDIC SURGEONS	"J Am Acad Orthop Surg"[jour]
JOURNAL OF THE AMERICAN PODIATRIC MEDICAL ASSOCIATION	"J Am Podiatr Med Assoc"[jour]

JOURNAL OF ARTHROPLASTY	"J Arthroplasty"[jour]
JOURNAL OF BACK AND MUSCULOSKELETAL REHABILITATION	"J Back Musculoskelet Rehabil"[jour]
JOURNAL OF BONE AND JOINT SURGERY AMERICAN VOLUME	"J Bone Joint Surg Am"[jour]
JOURNAL OF CHILDRENS ORTHOPAEDICS	"J Child Orthop"[jour]
JOURNAL OF EXPERIMENTAL ORTHOPAEDICS	"J Exp Orthop"[jour]
JOURNAL OF FOOT AND ANKLE RESEARCH	"J Foot Ankle Res"[jour]
JOURNAL OF FOOT ANKLE SURGERY	"J Foot Ankle Surg"[jour]
JOURNAL OF HAND SURGERY AMERICAN VOLUME	"J Hand Surg Am"[jour]
JOURNAL OF HAND SURGERY EUROPEAN VOLUME	"J Hand Surg Eur Vol"[jour]
JOURNAL OF HAND THERAPY	"J Hand Ther"[jour]
JOURNAL OF HIP PRESERVATION SURGERY	"J Hip Preserv Surg"[jour]
JOURNAL OF ISAKOS JOINT DISORDERS ORTHOPAEDIC SPORTS MEDICINE	"J ISAKOS"[jour]
JOURNAL OF KNEE SURGERY	"J Knee Surg"[jour]
JOURNAL OF ORTHOPAEDIC RESEARCH	"J Orthop Res"[jour]
JOURNAL OF ORTHOPAEDIC SCIENCE	"J Orthop Sci"[jour]
JOURNAL OF ORTHOPAEDIC SPORTS PHYSICAL THERAPY	"J Orthop Sports Phys Ther"[jour]
JOURNAL OF ORTHOPAEDIC SURGERY	"J Orthop Surg (Hong Kong)"[jour]
JOURNAL OF ORTHOPAEDIC SURGERY AND RESEARCH	"J Orthop Surg Res"[jour]
JOURNAL OF ORTHOPAEDIC TRANSLATION	"J Orthop Translat"[jour]
JOURNAL OF ORTHOPAEDIC TRAUMA	"J Orthop Trauma"[jour]
JOURNAL OF ORTHOPAEDICS AND TRAUMATOLOGY	"J Orthop Traumatol"[jour]
JOURNAL OF ORTHOPAEDICS	"J Orthop"[jour]
JOURNAL OF OSTEOPOROSIS	"J Osteoporos"[jour]
JOURNAL OF PEDIATRIC ORTHOPAEDICS PART B	"J Pediatr Orthop B"[jour]
JOURNAL OF PEDIATRIC ORTHOPAEDICS	"J Pediatr Orthop"[jour]
JOURNAL OF PHYSIOTHERAPY	"J Physiother"[jour]
JOURNAL OF PLASTIC SURGERY AND HAND SURGERY	"J Plast Surg Hand Surg"[jour]

JOURNAL OF SHOULDER AND ELBOW SURGERY	"J Shoulder Elbow Surg"[jour]
JOURNAL OF SPINAL DISORDERS TECHNIQUES	"J Spinal Disord Tech"[jour]
JOURNAL OF WRIST SURGERY	"J Wrist Surg"[jour]
JOR SPINE	"JOR Spine"[jour]
EKLEM HASTALIKLARI VE CERRAHISI JOINT DISEASES AND RELATED SURGERY	"Jt Dis Relat Surg"[jour]
JOINT DISEASES AND RELATED SURGERY	"Jt Dis Relat Surg"[jour]
KNEE SURGERY RELATED RESEARCH	"Knee Surg Relat Res"[jour]
KNEE SURGERY SPORTS TRAUMATOLOGY ARTHROSCOPY	"Knee Surg Sports Traumatol Arthrosc"[jour]
KNEE	"Knee"[jour]
MALAYSIAN ORTHOPAEDIC JOURNAL	"Malays Orthop J"[jour]
MINERVA ORTOPEDICA E TRAUMATOLOGICA	"Minerva Ortop Traumatol"[jour]
MLTJ MUSCLES LIGAMENTS AND TENDONS JOURNAL	"Muscles Ligaments Tendons J"[jour]
OPERATIVE TECHNIQUES IN ORTHOPAEDICS	"Oper Tech Orthop"[jour]
ORTHOPEDIC CLINICS OF NORTH AMERICA	"Orthop Clin North Am"[jour]
ORTHOPAEDIC JOURNAL OF SPORTS MEDICINE	"Orthop J Sports Med"[jour]
ORTHOPAEDIC NURSING	"Orthop Nurs"[jour]
ORTHOPEDIC REVIEWS	"Orthop Rev (Pavia)"[jour]
ORTHOPAEDIC SURGERY	"Orthop Surg"[jour]
ORTHOPAEDICS TRAUMATOLOGY SURGERY RESEARCH	"Orthop Traumatol Surg Res"[jour]
ORTHOPADE	"Orthopade"[jour]
ORTHOPEDICS	"Orthopedics"[jour]
OSTEOARTHRITIS AND CARTILAGE	"Osteoarthritis Cartilage"[jour]
PHYSICIAN AND SPORTSMEDICINE	"Phys Sportsmed"[jour]
PHYSICAL THERAPY	"Phys Ther"[jour]
PROSTHETICS AND ORTHOTICS INTERNATIONAL	"Prosthet Orthot Int"[jour]
REVISTA BRASILEIRA DE FISIOTERAPIA	"Rev Bras Fisioter"[jour]
SCOLIOSIS AND SPINAL DISORDERS	"Scoliosis Spinal Disord"[jour]

SICOT J	"SICOT J"[jour]
SKELETAL RADIOLOGY	"Skeletal Radiol"[jour]
SPINE	"Spine (Phila Pa 1976)"[jour]
SPINE DEFORMITY	"Spine Deform"[jour]
SPINE JOURNAL	"Spine J"[jour]
SPORTVERLETZUNG SPORTSCHADEN	"Sportverletz Sportschaden"[jour]
STRATEGIES IN TRAUMA AND LIMB RECONSTRUCTION	"Strategies Trauma Limb Reconstr"[jour]
WORLD JOURNAL OF ORTHOPEDICS	"World J Orthop"[jour]

Supplementary Materials S2. MEDLINE (PubMed) search strategy

#1 2012[pdat] OR 2022[pdat]

#2 "Asian J Endosc Surg"[jour]OR"Acta Chir Orthop Traumatol Cech"[jour]OR"Acta Orthop Belg"[jour]OR"Acta Orthop Traumatol Turc"[jour]OR"Acta Orthop"[jour]OR"Acta Ortop Bras"[jour]OR"Adv Exp Med Biol"[jour]OR"Adv Orthop"[jour]OR"Am J Sports Med"[jour]OR"Ann Jt"[jour] OR"Arch Bone Jt Surg"[jour]OR"Arch Orthop Trauma Surg"[jour]OR"Arch Osteoporos"[jour]OR"Arch Trauma Res"[jour]OR"Arthroplasty"[jour]OR"Arthrosc Tech"[jour]OR"Asia Pac J Sports Med Arthrosc Rehabil Technol"[jour]OR"Asian Spine J"[jour]OR"BMC Musculoskelet Disord"[jour]OR"Bone Joint J"[jour]OR"Bone Joint Res"[jour]OR"Bone Jt Open"[jour]OR"Bone Marrow Res"[jour]OR"Bonekey Rep"[jour]OR"Braz J Phys Ther"[jour]OR"Bull Hosp Jt Dis (2013)"[jour]OR"Cartilage"[jour]OR"Chin J Traumatol"[jour]OR"Chir Main"[jour]OR"Clin Biomech (Bristol, Avon)"[jour]OR"Clin J Sport Med"[jour]OR"Clin Med Insights Arthritis Musculoskelet Disord"[jour]OR"Clin Orthop Relat Res"[jour]OR"Clin Orthop Surg"[jour]OR"Clin Podiatr Med Surg"[jour]OR"Clin Spine Surg"[jour]OR"Connect Tissue Res"[jour]OR"Curr Orthop Pract"[jour]OR"Curr Rev Musculoskelet Med"[jour]OR"EFORT OPEN REVIEWS"[jour]OR"Eur Cell Mater"[jour]OR"Eur J Orthop Surg Traumatol"[jour]OR"Eur Spine J"[jour]OR"Foot Ankle Clin"[jour]OR"Foot Ankle Int"[jour]OR"Foot Ankle Spec"[jour]OR"Foot Ankle Surg"[jour]OR"Gait Posture"[jour]OR"Geriatr Orthop Surg Rehabil"[jour]OR"Global Spine J"[jour]OR"Hand Clin"[jour]OR"Hand Surg Rehabil"[jour]OR"Hip Int"[jour]OR"HSS J"[jour]OR"Indian J Orthop"[jour]OR"Injury"[jour]OR"Int J Shoulder Surg"[jour]OR"Int J Surg Open"[jour]OR"Int Orthop"[jour]OR"Isokinet Exerc Sci"[jour]OR"J Am Acad Orthop Surg Glob Res Rev"[jour]OR"J Am Acad Orthop Surg"[jour]OR"J Am Podiatr Med Assoc"[jour]OR"J Arthroplasty"[jour]OR"J Back Musculoskelet Rehabil"[jour]OR"J Bone Joint Surg Am"[jour]OR"J Child Orthop"[jour]OR"J Exp Orthop"[jour]OR"J Foot Ankle Res"[jour]OR"J Foot Ankle Surg"[jour]OR"J Hand Surg Am"[jour]OR"J Hand Surg Eur Vol"[jour]OR"J Hand Ther"[jour]OR"J Hip Preserv Surg"[jour]OR"J ISAKOS"[jour]OR"J Knee Surg"[jour]OR"J Orthop Res"[jour]OR"J Orthop

Sci"[jour]OR"J Orthop Sports Phys Ther"[jour]OR"J Orthop Surg (Hong Kong)"[jour]OR"J Orthop Surg Res"[jour]OR"J Orthop Translat"[jour]OR"J Orthop Trauma"[jour]OR"J Orthop Traumatol"[jour]OR"J Orthop"[jour]OR"J Osteoporos"[jour]OR"J Pediatr Orthop B"[jour]OR"J Pediatr Orthop"[jour]OR"J Physiother"[jour]OR"J Plast Surg Hand Surg"[jour]OR"J Shoulder Elbow Surg"[jour]OR"J Spinal Disord Tech"[jour]OR"J Wrist Surg"[jour]OR"JOR Spine"[jour]OR"Jt Dis Relat Surg"[jour]OR"Jt Dis Relat Surg"[jour]OR"Knee Surg Relat Res"[jour]OR"Knee Surg Sports Traumatol Arthrosc"[jour]OR"Knee"[jour]OR"Malays Orthop J"[jour]OR"Minerva Ortop Traumatol"[jour]OR"Muscles Ligaments Tendons J"[jour]OR"Oper Tech Orthop"[jour]OR"Orthop Clin North Am"[jour]OR"Orthop J Sports Med"[jour]OR"Orthop Nurs"[jour]OR"Orthop Rev (Pavia)"[jour]OR"Orthop Surg"[jour]OR"Orthop Traumatol Surg Res"[jour]OR"Orthopade"[jour]OR"Orthopedics"[jour]OR"Osteoarthritis Cartilage"[jour]OR"Phys Sportsmed"[jour]OR"Phys Ther"[jour]OR"Prosthet Orthot Int"[jour]OR"Rev Bras Fisioter"[jour]OR"Scoliosis Spinal Disord"[jour]OR"SICOT J"[jour]OR"Skeletal Radiol"[jour]OR"Spine (Phila Pa 1976)"[jour]OR"Spine Deform"[jour]OR"Spine J"[jour]OR"Sportverletz Sportschaden"[jour]OR"Strategies Trauma Limb Reconstr"[jour]OR"World J Orthop"[jour]OR

#3 (("Meta-Analysis as Topic"[MeSH] OR meta analy*[TIAB] OR metaanaly*[TIAB] OR "Meta-Analysis"[PT] OR "Systematic Review"[PT] OR "Systematic Reviews as Topic"[MeSH] OR systematic review*[TIAB] OR systematic overview*[TIAB] OR "Review Literature as Topic"[MeSH]) OR (cochrane[TIAB] OR embase[TIAB] OR psychlit[TIAB] OR psyclit[TIAB] OR psychinfo[TIAB] OR psycinfo[TIAB] OR cinahl[TIAB] OR cinhal[TIAB] OR "science citation index"[TIAB] OR bids[TIAB] OR cancerlit[TIAB]) OR (reference list*[TIAB] OR bibliograph*[TIAB] OR hand-search*[TIAB] OR "relevant journals"[TIAB] OR manual search*[TIAB]) OR (("selection criteria"[TIAB] OR "data extraction"[TIAB]) AND "Review"[PT])) NOT ("Comment"[PT] OR "Letter"[PT] OR "Editorial"[PT] OR ("Animals"[MeSH] NOT ("Animals"[MeSH] AND "Humans"[MeSH])))

#4 #1 AND #2 AND #3

Supplementary Materials S3. Epidemiological and reporting characteristics

Characteristics			Description example
Epidemiological characteristics			
		Number of authors	5
		Country of corresponding author	Japan

		Focus of review	Therapy (treatment/prevention), Epidemiology (prevalence, etiology), Diagnosis, Prognosis, Others
		SR category	Completely new, Update of prior SR, Newer scope than prior SR, Higher quality than prior SR, Limitations of primary studies only, Others
		Anatomical location	Spine, Pelvis, Upper limbs, Lower limbs, Others
		Common ICD-11 codes	Certain infections and parasitic diseases, Neoplasms, Diseases of the nervous system, Diseases of the circulatory system, Diseases of the musculoskeletal system or connective tissue, Injury, poisoning or certain other consequences of external causes, Others
		Number of included studies	8
		Number of included participants	400
		Economics assessment (i.e., costs) considered	Yes, No
		Meta-analysis performed	Pairwise MA, network MA, individual participant data MA, single-arm MA, Not performed
		Number of studies included in the largest meta-analysis	5
Reporting characteristics			
	Administrative information	SR protocol registration (e.g., PROSPERO) mentioned	Prospero, OSF, protocols.io, INPLASY, Not reported
		SR protocol mentioned	Protocol is publicly available, Protocol mentioned, but Not publicly available, Not reported
		Reporting guideline (e.g.,PRISMA) mentioned	PRISMA2009, PRISMA2020, MOOSE, QUOROM, MECIR, Not reported
		Cochrane handbook used	Yes, No

Search methods	Inclusion/exclusion criteria reported	Yes, No
	Number of databases searched (without trial registry)	5
	Trial registry (e.g., ClinicalTrials.gov) searched	Yes, No
	Eligible publication status	Both published and unpublished studies, Only published studies
Screening,extraction, and risk of bias assessment methods	All identified studies screened by at least two authors	Yes, No, Unclear
	All data extracted by at least two authors	Yes, No, Unclear
	Unpublished data acquired from original authors	Yes, No
	Study risk of bias/quality assessment by at least two authors	Yes, No, Unclear
	Study risk of bias/quality assessment tool used	Cochrane risk of bias tool, Cochrane risk of bias tool 2.0, MINORS, Newcastle-Ottawa Scale, Jadad scale, QUADAS or QUADAS-2, Reporting guideline (e.g., CONSORT), PEDro scal, MINORS, Not reported
Outcomes	Number of outcomes stated in the method	5, Not reported
	Primary outcome stated	Yes, No
	Statistical significance of effect estimate for primary outcome	Favourable and statistically significant, Favourable and statistically Nonsignificant, Unfavourable and statistically sgnificant, Unfavourable and statistically Nonsignificant, DirectioNof effect unclear, NA
	Magnitude of heterogeneity	<25%, 25 to <50%, 50 to <75%, 75% to 100%, Not reported

	(I2) in the meta-analysis for primary outcome	
	GRADE assessment reported in a summary of findings table or text	Yes, No
	Number of high certainty of evidence by GRADE assessment	0, NA
	moderate	0, NA
	low	1, NA
	very low	3, NA
Statistical methods	Risk of publication bias assessed (or intent to assess)	Formally assessed, Not assessed but authors planned, Not planned
	Subgroup analysis	Formally assessed, Not assessed but authors planned, Not planned
	Sensitivity analysis	Formally assessed, Not assessed but authors planned, Not planned
Others	Presence of COIs	Yes, No, Not reported
	Presence of funding	Yes, No, Not reported

Supplementary Materials S4. List of studies excluded from this review and reasons for exclusion

Reason for exclusion	Reference
Wrong study design	Tis JE, Karlin LI, Akbarnia BA, et al. Early onset scoliosis: modern treatment and results. *J Pediatr Orthop*. 2012;32(7):647-657.
Wrong study design	Riboh JC, Garrigues GE. Bone grafting in shoulder arthroplasty. *Orthopedics*. 2012;35(11):966-973.
Wrong study design	Sambunjak D, Franić M. Steps in the undertaking of a systematic review in orthopaedic surgery. *Int Orthop*. 2012;36(3):477-484.
Wrong study design	Benoist M, Boulu P, Hayem G. Epidural steroid injections in the management of low-back pain with radiculopathy: an update of their efficacy and safety. Eur Spine J. 2012 Feb;21(2):204-13
Wrong study design	Quality and usability of clinical assessments of static standing and sitting posture: A systematic review. J Back Musculoskelet Rehabil. 2022;35(2):223-238.

Wrong study design	Pearson NA, Tutton E, Joeris A, et al. A systematic review of outcome reporting in clinical trials of distal tibia and ankle fractures : the need for a core outcome set. *Bone Jt Open*. 2022;3(10):832-840.
Wrong study design	Norouzi-Barough L, Shirian S, Gorji A, Sadeghi M. Therapeutic potential of mesenchymal stem cell-derived exosomes as a cell-free therapy approach for the treatment of skin, bone, and cartilage defects. *Connect Tissue Res*. 2022;63(2):83-96.
Wrong study design	Li S, Wang S, Liu W, Zhang C, Song J. Current strategies for enhancement of the bioactivity of artificial ligaments: A mini-review. *J Orthop Translat*. 2022;36:205-215.
Wrong study design	Lacheta L, Braun S. Limited evidence for biological treatment measures for cartilage and tendon injuries of the shoulder. *Knee Surg Sports Traumatol Arthrosc*. 2022;30(4):1132-1137
Wrong study design	Morrow EM, Theologis T, Kothari A. Construction and validation of sham insoles used in clinical trials: A systematic review. *Prosthet Orthot Int*. 2022;46(2):121-133.
Wrong population	Cappello ZJ, Kasdan ML, Louis DS. Meta-analysis of imaging techniques for the diagnosis of complex regional pain syndrome type I. *J Hand Surg Am*. 2012;37(2):288-296.
Wrong population	Angel-Garcia D, Martinez-Nicolas I, Salmeri B, Monot A. Quality of Care Indicators for Hospital Physical Therapy Units: A Systematic Review. *Phys Ther*. 2022;102(2):pzab261.
Wrong population	Dave U, Kinderknecht J, Cheng J, Santiago K, Jivanelli B, Ling DI. Systematic review and meta-analysis of sex-based differences for concussion incidence in soccer. *Phys Sportsmed*. 2022;50(1):11-19.
Wrong population	Halpern AI, Jansen JAF, Giladi N, Mirelman A, Hausdorff JM. Does Time of Day influence postural control and gait? A review of the literature. *Gait Posture*. 2022;92:153-166.
Wrong population	Phu S, Lord SR, Sturnieks DL, Okubo Y. Impact of pathological conditions on postural reflex latency and adaptability following unpredictable perturbations: A systematic review and meta-analysis. *Gait Posture*. 2022;95:149-159.
Wrong population	O'Brien MW, Wu Y, Petterson JL, Bray NW, Kimmerly DS. Validity of the ActivPAL monitor to distinguish postures: A systematic review. *Gait Posture*. 2022;94:107-113.
Wrong population	Zhang Y, Li R, Miao X, Cheng LJ, Lau Y. Virtual motor training to improve the activities of daily living, hand grip, and gross motor function among children with cerebral palsy: Meta-regression analysis. *Gait Posture*. 2022;91:297-305.
Wrong population	Wang X, He Q, Ma L, You C. Comparison of different concentrations of hypertonic saline in patients with traumatic brain injury: Evidence from direct and indirect comparisons. *Injury*. 2022;53(11):3729-3735.
Wrong population	Mackenzie CF, Harris TE, Shipper AG, Elster E, Bowyer MW. Virtual reality and haptic interfaces for civilian and military open trauma surgery training: A systematic review. *Injury*. 2022;53(11):3575-3585.
Wrong population	Lei J, Gao GY, Jiang JY. Is management of acute traumatic brain injury effective? A literature review of published Cochrane

	Systematic Reviews. *Chin J Traumatol*. 2012;15(1):17-22.
Wrong population	Pinto RZ, Ferreira ML, Oliveira VC, et al. Patient-centred communication is associated with positive therapeutic alliance: a systematic review. *J Physiother*. 2012;58(2):77-87.
Wrong population	Li MD, Ahmed SR, Choy E, Lozano-Calderon SA, Kalpathy-Cramer J, Chang CY. Artificial intelligence applied to musculoskeletal oncology: a systematic review. *Skeletal Radiol*. 2022;51(2):245-256.
Wrong population	Xie R, Zhong A, Wu J, Cen Y, Chen J. Could hyperbaric oxygen be an effective therapy option for pathological scars? A systematic review and meta-analysis [published online ahead of print, 2022 May 18]. *J Plast Surg Hand Surg*. 2022;1-6.

Supplementary Materials S5. Comparison of the reporting characteristics of systematic reviews in orthopedic journals published in 2012 and 2022, stratified by focus of systematic reviews

Characteristic	Category	Therapy		Epidemiology		Diagnosis		Prognosis	
		2012 (n = 113)	2022 (n = 143)	2012 (n = 12)	2022 (n = 19)	2012 (n = 21)	2022 (n = 7)	2012 (n = 19)	2022 (n = 20)
SR protocol registration (e.g., PROSPERO) mentioned	Not reported	100%	↓ 68%	100%	↓ 53%	95%	↑ 100%	89%	↓ 60%
	Prospero	0%	↑ 29%	0%	↑ 42%	5%	↓ 0%	0%	↑ 35%
	The others	0%	↑ 3%	0%	↑ 5%	0%	= 0%	11%	↓ 5%
Reporting guideline (e.g.,PRISMA) mentioned	Not reported	77%	↓ 10%	67%	↓ 26%	57%	↓ 14%	42%	↓ 15%
	PRISMA 2009	12%	↑ 41%	17%	↑ 26%	29%	↑ 57%	32%	↑ 60%
	PRISMA 2020	0%	↑ 14%	0%	↑ 11%	0%	= 0%	0%	↑ 5%
	PRISMA extension and the other PRISMA-related	6%	↑ 27%	8%	↑ 37%	10%	↑ 29%	26%	↓ 15%
Cochrane handbook used		21%	↓ 16%	8%	↓ 11%	14%	↓ 0%	5%	↑ 10%
Inclusion/exclusion criteria reported		73%	↑ 94%	100%	↓ 95%	95%	↓ 86%	100%	= 100%

Number of databases searched (without trial registry)		3 (2-5)	↑	4 (3-4)	3 (2-3.5)	=	3 (3-4)	4 (3-6)	↓	3 (1-4)	3 (2-5)	=	3 (3-4)
Trial registry (e.g., ClinicalTrials.gov) searched		5%	↑	9%	0%	↑	11%	10%	↑	14%	5%	=	5%
All identified studies screened by at least two authors		58%	↑	84%	25%	↑	79%	67%	↓	57%	53%	↑	90%
All data extracted by at least two authors		40%	↑	53%	8%	↑	37%	29%	↑	57%	26%	↑	55%
Unpublished data acquired from original authors		21%	↓	6%	8%	↑	11%	19%	↓	0%	5%	↓	0%
Study risk of bias/quality assessment by at least two authors		48%	↓	47%	8%	↑	32%	33%	↑	57%	42%	↑	65%
Study risk of bias/quality assessment tool used	Not reported	38%	↓	15%	75%	↓	47%	10%	↑	29%	47%	↓	0%
	Cochrane risk of bias tool	16%	↓	10%	0%	↑	11%	5%	↓	0%	5%	=	5%
	Cochrane risk of bias tool 2.0	0%	↑	8%	0%	=	0%	0%	=	0%	0%	=	0%
	MINORS	1%	↑	17%	0%	↑	32%	0%	=	0%	0%	=	0%
	Newcastle-Ottawa Scale	1%	↑	7%	8%	↓	0%	5%	↓	0%	21%	↓	5%
Number of outcomes stated in the method		4 (2-7)	↓	3 (2-5)	2 (1-3.5)	=	2 (1-3)	2.5 (1-5)	↓	1 (1-4)	1.5 (1-2)	↓	1 (1-4)
Primary outcome stated		24%	↑	42%	0%	↑	58%	14%	↑	71%	21%	↑	65%
Statistical significance of effect estimate for primary	Not reported	58%	↓	54%	NA		NA	81%	↓	71%	84%	↓	50%

outcome	Favourable and statistically significant	17%	↑	22%	NA		NA	5%	↓	0%	5%	↑	15%
	Favourable and statistically nonsignificant	14%	↑	15%	NA		NA	0%	=	0%	5%	↓	0%
	Unfavourable and statistically significant	6%	↓	3%	NA		NA	0%	=	0%	5%	↑	20%
	Unfavourable and statistically nonsignificant	4%	↑	6%	NA		NA	0%	=	0%	0%	↑	10%
	Direction of effect unclear	0%	=	0%	NA		NA	0%	=	0%	0%	=	0%
Magnitude of heterogeneity (I2) in the MAs for primary outcome	Not reported	9%	↑	50%	92%	↑	95%	76%	↓	57%	74%	↓	50%
	<25%	14%	↑	16%	0%	↑	5%	14%	↓	0%	5%	↑	25%
	25 to <50%	7%	↑	8%	0%	=	0%	0%	=	0%	21%	↓	0%
	50 to <75%	10%	↓	7%	0%	=	0%	0%	↑	29%	0%	↑	5%
	75% to 100%	6%	↑	19%	8%	↓	0%	5%	↑	14%	0%	↑	20%
GRADE assessment reported in a summary of findings table or text		8%	↑	10%	0%	↑	5%	0%	=	0%	16%	↓	5%
Proportion of certainty of evidence by GRADE assessment	High certainty of evidence	0%	↑	2%	0%	=	0%	0%	=	0%	5%	↓	0%
	Moderate certainty of evidence	3%	↑	5%	0%	=	0%	0%	=	0%	11%	↓	0%
	Low certainty of evidence	4%	↑	5%	0%	=	0%	0%	=	0%	11%	↓	5%
	Very low certainty of evidence	6%	↓	3%	0%	↑	5%	0%	=	0%	0%	=	0%
Risk of publication bias	Not planned	81%	↓	79%	92%	↑	100%	95%	↓	57%	74%	↑	75%

assessed (or intent to assess)	Formally assessed	17%	↑ 20%	8%	↓ 0%	5%	↑ 43%	26%	↓ 25%
	Not assessed but authors planned	2%	↓ 1%	0%	= 0%	0%	= 0%	0%	= 0%
Subgroup analysis	Not planned	76%	↑ 81%	92%	↑ 100%	86%	↓ 71%	100%	↓ 65%
	Formally assessed	21%	↓ 17%	8%	↓ 0%	10%	↑ 29%	0%	↑ 30%
	Not assessed but authors planned	3%	↓ 1%	0%	= 0%	0%	= 0%	0%	= 0%
Sensitivity analysis	Not planned	83%	↑ 89%	92%	↑ 95%	100%	↓ 86%	95%	= 95%
	Formally assessed	14%	↓ 11%	8%	↓ 5%	0%	↑ 14%	5%	= 5%
	Not assessed but authors planned	3%	↓ 0%	0%	= 0%	0%	= 0%	0%	= 0%
Presence of COIs	No	62%	↑ 82%	50%	↑ 74%	67%	↑ 100%	58%	↑ 65%
	Yes	20%	↓ 17%	25%	↓ 16%	24%	↓ 0%	32%	↑ 35%
	Not reported	18%	↓ 1%	25%	↓ 11%	10%	↓ 0%	11%	↓ 0%
Presence of funding	No	41%	↑ 57%	25%	↑ 58%	24%	↑ 57%	26%	↑ 55%
	Yes	21%	↑ 29%	25%	↑ 26%	29%	↑ 29%	42%	↓ 40%
	Not reported	38%	↓ 14%	50%	↓ 16%	48%	↓ 14%	32%	↓ 5%

SR, systematic review; MA, meta-analysis; GRADE, Grading of Recommendations, Assessment, Development, and Evaluations; COI, conflict of interest; NA, not applicable. Data was presented as number (percent) or median (IQR). Direction of change in 2012 versus 2022 means as follows; ↑, increase; ↓, decrease; =, no change.

Supplementary Materials S6. Odds ratio association reporting characteristics and protocol registration

Reporting characteristics	Protocol registration	Non- protocol registration	Odds ratio (95% CI)
Reporting guideline mentioned	66/71	155/289	11.41 (4.47, 29.16)
Cochrane handbook used	15/71	42/289	1.58 (0.82, 3.04)
Inclusion/exclusion criteria reported	70/71	277/289	3.03 (0.39, 23.72)

Trial registry searched	12/71	14/289	4.0 (1.76, 9.08)
Screened by at least two authors	59/71	196/289	2.33 (1.2, 4.55)
Risk of bias/quality assessment tool used	66/71	155/289	11.41 (4.47, 29.16)
Unpublished data acquired	8/71	32/289	1.02 (0.45, 2.32)
Primary outcome stated	33/71	92/289	1.86 (1.1, 3.15)
Statistical significance of effect estimate for primary outcome	14/71	60/289	0.94 (0.49, 1.8)
GRADE assessment reported	9/71	20/289	1.95 (0.85, 4.49)
Publication bias assessed	16/71	46/289	1.54 (0.81, 2.91)
Subgroup analysis assessed	10/71	50/289	0.78 (0.38, 1.63)
Sensitivity analysis assessed	7/71	30/289	0.94 (0.4, 2.25)

Supplementary Materials S7. Odds ratio association reporting characteristics and self-reported use of PRISMA

Reporting characteristics	PRISMA	Non-PRISMA	Odds ratio (95% CI)
Cochrane handbook used	24/105	7/15	0.34 (0.11, 1.03)
Inclusion/exclusion criteria reported	103/105	15/15	1.34 (0.06, 29.14)
Trial registry searched	9/105	2/15	0.61 (0.12, 3.14)
Screened by at least two authors	82/105	12/15	0.89 (0.23, 3.43)
Risk of bias/quality assessment tool used	105/105	6/15	308.38 (16.11, 5904.48)
Unpublished data acquired	12/105	4/15	0.35 (0.1, 1.29)
Primary outcome stated	41/105	8/15	0.56 (0.19, 1.66)
Statistical significance of effect estimate for primary outcome	29/105	5/15	0.76 (0.24, 2.42)
GRADE assessment reported	13/105	1/15	1.98 (0.24, 16.32)

Publication bias assessed	21/105	7/15	0.29 (0.09, 0.88)
Subgroup analysis assessed	19/105	4/15	0.61 (0.17, 2.12)
Sensitivity analysis assessed	17/105	15/15	0.01 (0, 0.11)
